# No difference in extra-axial cerebrospinal fluid volumes across neurodevelopmental and psychiatric conditions in later childhood and adolescence

**DOI:** 10.1186/s11689-023-09477-x

**Published:** 2023-04-01

**Authors:** Madeline Peterson, Christopher Whetten, Anne M. Clark, Jared A. Nielsen

**Affiliations:** 1 Department of Psychology, Brigham Young University, Provo, UT 84602 USA; 2 Neuroscience Center, Brigham Young University, Provo, UT 84604 USA

**Keywords:** Autism spectrum disorder, Brain development, Cerebrospinal fluid, Extra-axial cerebrospinal fluid, MRI

## Abstract

**Background:**

While autism spectrum disorder has been associated with various organizational and developmental aberrations in the brain, an increase in extra-axial cerebrospinal fluid volume has recently garnered attention. A series of studies indicate that an increased volume between the ages of 6 months and 4 years was both predictive of the autism diagnosis and symptom severity regardless of genetic risk for the condition. However, there remains a minimal understanding regarding the specificity of an increased volume of extra-axial cerebrospinal fluid to autism.

**Methods:**

In the present study, we explored extra-axial cerebrospinal fluid volumes in children and adolescents ages 5–21 years with various neurodevelopmental and psychiatric conditions. We hypothesized that an elevated extra-axial cerebrospinal fluid volume would be found in autism compared with typical development and the other diagnostic group. We tested this hypothesis by employing a cross-sectional dataset of 446 individuals (85 autistic, 60 typically developing, and 301 other diagnosis). An analysis of covariance was used to examine differences in extra-axial cerebrospinal fluid volumes between these groups as well as a group by age interaction in extra-axial cerebrospinal fluid volumes.

**Results:**

Inconsistent with our hypothesis, we found no group differences in extra-axial cerebrospinal fluid volume in this cohort. However, in replication of previous work, a doubling of extra-axial cerebrospinal fluid volume across adolescence was found. Further investigation into the relationship between extra-axial cerebrospinal fluid volume and cortical thickness suggested that this increase in extra-axial cerebrospinal fluid volume may be driven by a decrease in cortical thickness. Furthermore, an exploratory analysis found no relationship between extra-axial cerebrospinal fluid volume and sleep disturbances.

**Conclusions:**

These results indicate that an increased volume of extra-axial cerebrospinal fluid may be limited to autistic individuals younger than 5 years. Additionally, extra-axial cerebrospinal fluid volume does not differ between autistic, neurotypical, and other psychiatric conditions after age 4.

## Introduction

Autism spectrum disorder (ASD) is a heterogeneous neurodevelopmental condition typified by deficits in social communication and the presence of restricted repetitive behaviors (*Diagnostic and Statistical Manual-5* [DSM-5]; [1]). As a neurodevelopmental condition, ASD is associated with aberrant trajectories in social, cognitive, and physiological development. For example, one well-replicated finding is that of an increased brain volume [2–5] and head size in comparison with typically developing children [6–9]. Likewise, task-based and resting state functional connectivity research indicates that autistic children may undergo a transition from hypoconnectivity [10–12] to hyperconnectivity [13–15], with puberty as the proposed time of transition [16].

Beyond these atypical patterns in neurodevelopment, intervention can alter the course of social and cognitive autistic development. For instance, previous work [17] has indicated that earlier intervention results in improved behavioral outcomes (although the nature of those outcomes is debated, see [18]). In order to facilitate that earlier intervention, investigators have called for the identification of biomarkers that could assist in the diagnosis of ASD [19]. However, in order for such biomarkers to emerge, specificity to the condition of interest must be established. This is particularly important when there is significant overlap in symptomatology and underlying etiology, as well as high rates of comorbidity, between the target condition and other conditions.

### Shared symptomatology

One challenge to distinguishing between ASD and other neurodevelopmental or psychiatric conditions such as generalized anxiety disorder (GAD), social anxiety disorder, attention-deficit hyperactivity disorder (ADHD), and specific learning disorders is the significant overlap of traits, symptoms, and characteristics. Autism spectrum disorder is defined by characteristic deficits in social behavior and communication as well as repetitive behaviors and interests, with the understanding that there is significant variation in severity, clinical presentation, and level of functioning [1]. The overlap in syndromal presentation in areas such as social interaction or cognitive deficit can make it difficult to discriminate between ASD and other conditions. For example, the DSM-5 principally defines social anxiety disorder by social avoidance and fear of negative evaluation, which could be mistaken for the social communication deficit present in ASD [1]. Autism spectrum disorder and generalized anxiety disorder present considerable symptomatic overlap such as with excessive worry, self-consciousness, difficulty identifying and expressing emotion, obsessive behavior, trouble with concentration, a compulsive need for routines and continuity, agitated behavior, and phobias [20]. Findings from studies comparing individuals with ADHD and ASD diagnoses suggest a similar overlap. For example, one comparison study was unable to identify a reliable difference in cognitive abilities between autistic individuals and individuals with ADHD on measures such as attention and arousal, response inhibition, working memory, interference control, and processing speed [21].

In a study examining rates of comorbid psychiatric disorders in autistic children, 70% of participants had at least one comorbid disorder, with the two most prevalent of these comorbidities being social anxiety disorder (29.2%) and ADHD (28.1%) [22]. Additionally, research has reported that 39.6% of adolescent autistic individuals qualified for at least one comorbid anxiety disorder under the *Diagnostic and Statistical Manual of Mental Disorders*, Fourth Edition [23]. However due to the complexity of syndromal overlap, the estimation of comorbid prevalence is not always so clean. For example, comorbid rates of ASD and reading disorder (a specific learning disorder) can vary from as low as 6% to up to 30% [24]. In parallel, it is estimated that anywhere between 30 and 80% of autistic individuals also qualify for a diagnosis of ADHD, and that ASD is present in 20–50% of individuals with ADHD [25, 26].

### A potential biomarker

Essential to the diagnosis of these conditions is specificity to the condition at hand, both with regard to neural mechanisms as well as for other biomarkers. Effective biomarkers can distinguish between conditions with high rates of co-occurrence and similar symptomology.

One candidate biomarker, an increased volume of extra-axial cerebrospinal fluid (EA-CSF), is amassing credibility due to both theoretical significance and empirical evidence. Cerebrospinal fluid (CSF) plays an essential role in the development of the brain, maintaining homeostasis within the cerebral interstitial fluid, regulating the electrolyte balance, and eliminating catabolites [27]. Furthermore, in early development, CSF distributes growth factors which signal progenitor cells to proliferate into immature neurons, which later migrate to different areas of the cerebral cortex [28]. Together, these key roles place CSF in a position to impact neurodevelopment.

In addition to the extensive role of CSF in development, empirical evidence linking EA-CSF volume to ASD has come forward. EA-CSF volume has been operationalized as the amount of cerebrospinal fluid surrounding the dorsal convexity of the cerebral cortex in the subarachnoid space and was found to be increased in infants and children ages 6 months to 4 years who would later be diagnosed with autism. This relationship was documented in three separate samples and studies [29–31]. In each of these studies, an increased volume of EA-CSF preceded the onset and severity of the autism diagnosis and symptoms, regardless of genetic risk [29–31]. Interestingly, in an accelerated, multi-cohort longitudinal study of participants 3–42 years, no difference in EA-CSF volume between the ASD and control groups was found, suggesting that EA-CSF volume may normalize after age 4 in ASD [32]. Despite this evidence, temporal precedence alone cannot establish causality [33]. Among other elements, specificity must also be established. Thus, in their 2020 study, Murphy and colleagues [34] examined EA-CSF volumes in 1–2 years old typically developing children and children at risk for schizophrenia, finding no difference in EA-CSF volume between the two groups. Given the symptom overlap and frequent co-occurrence of anxiety disorders, ADHD, and other neurodevelopmental conditions with ASD, it is important to determine if an increased volume of EA-CSF is specific to ASD in comparison with these conditions.

### EA-CSF volume and sleep disturbances

In addition to understanding the specificity of an increased volume of EA-CSF to ASD, it is important to identify the potential ramifications of this disruption for specific behaviors. Highly prevalent to psychopathology and found in almost every major psychiatric condition are sleep disturbances [35]. In ASD specifically, there is an increased incidence of sleep problems compared with typical development that begin before age 2 and persist across the lifespan (reviewed in [36]). Sleep disturbances are also documented in individuals with anxiety and related conditions and may predict the development of an anxiety disorder [37, 38].

One potential mechanism behind this relationship between sleep disturbances and psychopathology may be CSF flow. CSF plays a crucial role in maintaining cerebral homeostasis, and its contents can reflect specific brain states, including satiety, sleep, and disease (reviewed in [39]). During natural sleep and anesthesia, the flow of CSF is increased, and there is an increased rate of beta-amyloid clearance and other neuropeptides during sleep compared to wakefulness in an animal model [40]. In humans, sleep results in greater glymphatic clearance in comparison with awake states [41] and a night of sleep deprivation [42]. Thus, disturbances to sleep appear to result in changes to CSF exchange and flow rates, impacting neuronal performance and potentially contributing to pathology. This conjecture was evidenced by Shen et al. [31], which demonstrated that a subgroup of autistic participants with high EA-CSF volumes (defined as 1.5 standard deviations or more above the mean) had more sleep problems than the other autistic and typically developing participants. However, it is unknown if this relationship between EA-CSF volume and sleep disturbances in autism continues into later childhood and adolescence.

### EA-CSF volume and cortical thickness

Beyond examining the consequences of an increased volume of EA-CSF, it is also important to consider possible mechanisms. Due to the close anatomical proximity of EA-CSF to the cerebrum, changes in cortical thickness are a reasonable candidate. Such changes to cortical thickness may occur during synaptic pruning events, such as that during adolescence when there is a well-documented decrease in mean cortical thickness [43–45]. Such a decrease in cortical thickness could create a deficiency in the subarachnoid space which could then be filled with an increase in the volume of EA-CSF as postulated by Peterson et al. [32]. This relationship between a decrease in mean cortical thickness and an increase in EA-CSF volume has been evidenced in a longitudinal sample of participants 3 to 42 years of age [32]. However, it remains to be seen if this finding can be replicated in a different sample.

### Specific hypotheses

We hypothesize that the volume of EA-CSF will be significantly increased in individuals with ASD compared to other individuals with neurodevelopmental or psychiatric conditions and healthy controls, similar to previous findings [29–31, 34]. We also hypothesize that there will be an effect of age on EA-CSF volume in ASD and typical development comparable to that found in [32]. Additionally, it is hypothesized that there will be a positive relationship between sleep problems and EA-CSF volume across all groups, as suggested by [31]. Finally, we hypothesize that there will be a negative relationship between EA-CSF volume and cortical thickness across all groups as previously demonstrated [32]. With a cross-sectional dataset of 446 images from children and adolescents 5–21 years old, we tested these hypotheses.

## Materials and methods

### Participants

The current study is an analysis of structural magnetic resonance imaging (MRI) data that has previously been collected at various research sites in accordance with Healthy Brain Network protocols; see [46]. This dataset has previously been reported on by various teams including Mihailov et al. [47], Nentwich et al. [48], and Palumbo et al. [49]. Since data collection is ongoing, we used MRI and phenotypic data available as of 12/1/2020, and this includes data releases 1–7. This particular analysis has been preregistered on the Open Science Framework (https://osf.io/kbh73), and all codes employed can be found on GitHub (https://github.com/peter3200/HBN_Project). Deviations from the preregistration included the approach to fencing outliers in EA-CSF volume, the addition of two covariates in the main model, and an exploratory analysis examining the relationship between EA-CSF volume and sleep disturbances. Participants initially included 2196 individuals, with 2034 total T1-weighted images. After a quality-control process (described in a later section) and the implementation of the exclusion criteria, the final number of scans included in the analysis was 446.

**Fig. 1:**
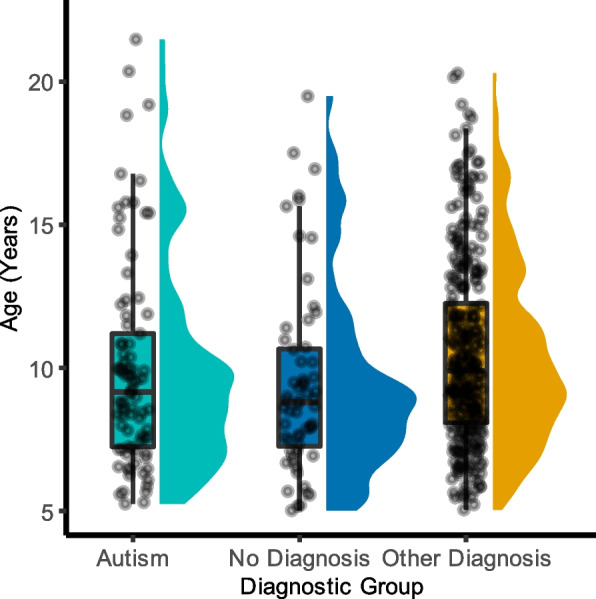
Participant age at scan. Following the quality-control procedures, participants included 85 autistic individuals (67 males, 18 females), 60 individuals with no diagnosis (35 males, 25 females), and 301 individuals with a diagnosis other than ASD (179 males, 122 females). ASD mean age was 9.98 years, range 5.25–21.48 years; Other Diagnosis mean age was 10.48 years, range 5.04–20.29 years; No Diagnosis mean age was 9.46 years, range 5.02–19.49 years; overall mean age was 10.25 years, range 5.02–21.48 years

As indicated in Alexander et al. [46], clinical diagnoses were determined by licensed clinicians using the computerized web-based version of the Schedule for Affective Disorders and Schizophrenia—Children’s version [50]. Upon completion of this and other assessments conducted during study participation, clinically synthesized diagnoses were generated by the clinical team. For the purposes of this analysis, diagnostic group was determined as follows: participants with an ASD diagnosis were included only in the ASD group (*N* = 85; 67 males, 18 females), participants with “No Diagnosis Given” were included only in the No Diagnosis group (*N* = 60; 35 males, 25 females), and participants with diagnoses other than ASD or No Diagnosis were included in the Other Diagnosis group (*N* = 301; 179 males, 122 females). Furthermore, diagnostic groups other than ASD were included in the analysis within the overall Other Diagnosis group if there were 50 or more T1w images available for that specific diagnosis. Those diagnoses included the following: attention-deficit/hyperactivity disorder-combined, attention-deficit/hyperactivity disorder-inattentive, generalized anxiety disorder, social anxiety disorder, and specific learning disorder-reading. Additional demographic information can be found in Table 1. In summary, ASD mean age was 9.98 years, range 5.25–21.48 years; Other Diagnosis mean age was 10.48 years, range 5.04–20.29 years; No Diagnosis mean age was 9.46 years, range 5.02–19.49 years; and overall mean age was 10.25 years, range 5.02–21.48 years (see Fig. 1). Furthermore, using the criterion of an average full-scale intelligence quotient (IQ) score less than or equal to 79 to operationalize low verbal and cognitive performance [51], it was found that 50 scans came from participants with low verbal and cognitive performance (18 ASD, 32 Other Diagnosis). Additionally, 319 scans came from 50 ASD participants, 45 No Diagnosis participants, and 224 Other Diagnosis participants with high verbal and cognitive performance. Of the ASD participants included in the analysis, Seventy-eight had a comorbid condition (see Table 2 for additional details on comorbid diagnoses in ASD).

**Table 1:** Demographics

	Autism, *N* = 85		Other diagnosis, *N* = 301		No diagnosis, *N* = 60		Group comparison^a^	
	Mean (SD)	Range	Mean (SD)	Range	Mean (SD)	Range	*χ*^2^ or *F*	*p*
Biological sex^b^ (male/female)	67 M	18 F	179 M	122 F	35 M	25 F	11.3	< .05
Age (years)	9.98 (3.64)	5.25–21.48	10.48 (3.21)	5.04–20.29	9.46 (3.31)	5.02–19.49	2.76	.06
Full-scale IQ^c^	97.15 (21.78)	51–147	98.66 (15.79)	59–143	112.18 (13.25)	85–145	13.64	< .001
Social Responsiveness Scale total (raw)^d^	80.27 (27.42)	18–147	50.29 (28.07)	3–146	28.06 (16.6)	4–97	63.87	< .001
Sleep Disturbance Scale for Children total (raw)^e^	41.48 (10.99)	27–82	42.03 (10.93)	26–84	36.68 (7.07)	26–59	4.82	< .01

^a^Group comparisons were initially conducted on each demographic variable using a chi-squared test (biological sex only) or a one-way ANOVA. ^b^Post hoc chi-squared tests indicated significant group differences in biological sex between the Autism and No Diagnosis groups (*χ*^2^ = 6.13, *p* = .01) and between the Autism and Other Diagnosis groups (*χ*^2^ = 9.92, *p* < .01). ^c^Full-scale IQ (WISC): Autism *N* = 68, Other Diagnosis *N* = 256, No Diagnosis *N* = 45. Post hoc, Bonferroni-adjusted *t*-tests indicated significant group differences in full-scale IQ between the Autism and No Diagnosis groups (*p* < .001) as well as the Other Diagnosis and No Diagnosis groups (*p* < .001). ^d^Social Responsiveness Scale: Autism *N* = 77, Other Diagnosis *N* = 286, No Diagnosis *N* = 52. Post hoc, Bonferroni-adjusted *t*-tests indicated significant group differences in the Social Responsiveness Scale total score between all groups (*p* < .001). ^e^Sleep Disturbance Scale: Autism *N* = 69, Other Diagnosis *N* = 254, No Diagnosis *N* = 44. Post hoc, Bonferroni-adjusted *t*-tests indicated a significant group difference in the Sleep Disturbance Scale total score between the Other Diagnosis and No Diagnosis groups (*p* < .01)

**Table 2:** Comorbid conditions in ASD^a^

	ADHD combined type	ADHD inattentive type	Generalized anxiety disorder	Social anxiety disorder	Specific learning disorder-reading
ADHD combined type	40	0	5	0	6
ADHD inattentive type	-	21	3	3	1
Generalized anxiety disorder	-	-	8	1	2
Social anxiety disorder	-	-	-	6	0
Specific learning disorder-reading	-	-	-	-	9

^a^Each table row indicates the number of ASD participants with a comorbid diagnosis. Columns indicate a third comorbid diagnosis. For example, the first column of the first row represents the number of participants diagnosed with ASD and ADHD combined type (*N* = 40). The third column of the first row represents the number of participants diagnosed with ASD, ADHD combined type, and generalized anxiety disorder (*N* = 5)

### Behavioral measures

As indicated in Table 1, the Wechsler Intelligence Scale for Children-V (WISC; [52]), Social Responsiveness Scale-2 [53], and Sleep Disturbances Scale for Children [54] were administered to participants as a part of the Healthy Brain Network study protocol. The WISC was administered to participants 6–17 years old and is a measure of cognitive function in children and adolescents. The WISC-V is test-retest reliable and internally consistent, with a corrected test-retest reliability coefficient for the full-scale IQ score at 0.92 and estimated internal consistency for the overall normative sample using split-half reliability at 0.96 [55]. Second, the Social Responsiveness Scale is a quantitative measure of the various dimensions of interpersonal behavior, communication, and repetitive/stereotypic behavior characteristic of ASD, and this measure was administered to the parents of all participants. The 3-month test-retest reliability for this measure is 0.88 in clinical subjects [53]. Additional psychometric properties of the Social Responsiveness Scale have been previously reported (see [56, 57]). Finally, the Sleep Disturbances Scale for Children was administered to the parents of all participants, and a higher total score indicates a greater sleep disturbance. Bruni et al. [54] conducted a psychometric evaluation of the Sleep Disturbances Scale and found an internal consistency ranging from 0.71 to 0.79 and a test-retest reliability of 0.71.

### Image acquisition procedure

This dataset is composed of 446 T1-weighted structural MRI scans collected at three different sites in accordance with the Chesapeake Institutional Review Board (https://www.chesapeakeirb.com/). Two-hundred sixty-eight scans were collected in the Rutgers University Brain Imaging Center on a 3T Tim Trio MRI scanner, and 178 scans were collected at the CitiGroup Cornell Brain Imaging Center on a 3T Siemens Prisma scanner. Furthermore, no scans collected at the mobile trailer facility (equipped with a 1.5T Siemens Avanto system with 45 mT/m gradients) survived the quality-control protocol. Additional details regarding scan sequences and other parameters have been reported elsewhere ([46]; http://fcon_1000.projects.nitrc.org/indi/cmi_healthy_brain_network/MRI%20Protocol.html).

### Image processing

EA-CSF was defined as CSF within the subarachnoid space surrounding the dorsal convexity (thus excluding all spinal and ventricular CSF), with a ventral boundary at the plane of the anterior and posterior commissures [29–31]. In order to derive the volume of EA-CSF from these scans, we implemented a neuroimaging pipeline that included the following steps. To begin, the data underwent preprocessing via the ANTs tool N4BiasFieldCorrection [58]. This step is necessary to correct for bias field signal, which can result in non-uniformities in image intensity. After this step, resampling via the package c3d [59] was undertaken since images acquired at the Staten Island facility (the 1.5T Siemens Avanto system) had different voxel dimensions (1.0 mm by 1.0 mm by 1.0 mm) than the other two sites (0.8 mm by 0.8 mm by 0.8 mm). All images were resampled to 1.0-mm isotropic voxels.

Following these preprocessing steps, we processed the structural images with the Automatic Extra-axial Cerebrospinal Fluid (Auto EACSF) pipeline version 1.7.7 [30]. The pipeline registers, skullstrips, and then segments each T1-weighted image. After segmentation, with a ventricle mask and template as input, the tool generates a progressive series of images such that any ventricular or cistern CSF is gradually eradicated from one image to the next. After inspecting the intermediate output files, it was determined that the output file with the suffix “MID02” was most accurate at approximating EA-CSF (later output files such as the “QCistern” file tended to be overaggressive in stripping away EA-CSF). Finally, the computational morphometry toolkit was employed to calculate the number of CSF voxels in each MID02 file. See GitHub (https://github.com/peter3200/HBN_Project) for all code employed to run preprocessing and pipeline applications.

The estimated total intracranial volume (eTIV) for each participant was extracted as a control measure since a significant difference in total brain volume was found between the ASD and control groups in previous work by Shen and colleagues [29–31]. To extract eTIV, images were automatically processed with the FreeSurfer analysis suite version 7.1.1, which is documented and freely available for download online (http://surfer.nmr.mgh.harvard.edu/). Additionally, mean cortical thickness was also extracted since work by Peterson et al. [32] demonstrated a negative relationship between EA-CSF volume and mean cortical thickness. The technical details of these procedures are described in prior publications [60–73]. Freesurfer morphometric procedures have been demonstrated to show good test-retest reliability across scanner manufacturers and across field strengths [69, 72].

### Visual quality control

Both the raw T1-weighted images and Auto EACSF output images for all participants underwent visual quality control, such that four raters each rated half of the dataset. The raters trained on a set of separate datasets and after achieving an intraclass correlation greater than 0.90 went on to rate the study images.

Raw T1-weighted images were assessed for quality and the presence of motion artifacts, using a previously described four-scale rating system [74]. The output images from Auto EACSF were assessed for the quality of segmentation using the rating scale and standard images developed by [34]. Thus, two scores were assigned to each scan: a structural quality score and a segmentation quality score. Only scans with a structural score less than three and a segmentation score less than two (no abnormalities in segmentation or minor under- or overestimation of EA-CSF in one region) were included in this study [34]. The raters were blinded to the identities of the images, and the overall interrater reliability was 0.95 before discrepancies in ratings were resolved. If raters differed in their ratings, a third rater was brought in to reconcile the ratings.

**Fig. 2:**
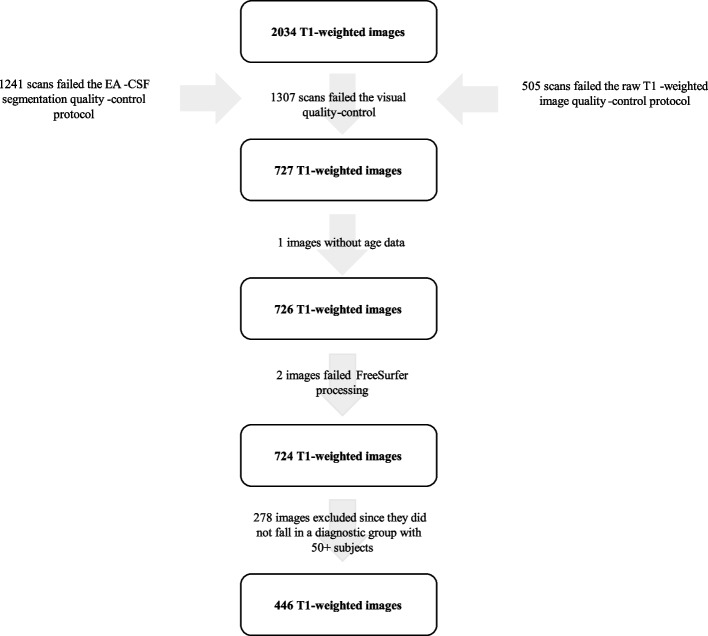
Number of scans included in the analysis. Following the quality-control protocols and diagnostic group determination procedure (as outlined in the study pre-registration), 446 total T1-weighted images were included in the analysis

The initial dataset was comprised of 2034 scans (*ASD*: 243, Other Diagnosis: 1612, No Diagnosis: 179; see Fig. 2 for the total number of scans included in the analysis). Of these, 1241 scans failed the segmentation protocol (*ASD*: 151, Other Diagnosis: 980, No Diagnosis: 110), and 505 scans failed the raw T1-weighted image protocol (*ASD*: 69, Other Diagnosis: 402, No Diagnosis: 34). The EA-CSF segmentation rating was not significantly associated with the Social Responsiveness Scale score (*r* = 0.05, *t*(1458) = 1.94, *p* = .05), but it was significantly correlated with full-scale IQ (*r* = −0.07, *t*(1257) = −2.67, *p* = .008), indicating that as full-scale IQ decreased, the quality of the scan also decreased (resulting in a greater quality-control rating).

### Statistical analysis

The analysis began with the fencing of outliers in EA-CSF volume, which, in a slight deviation from the preregistration, were identified for each year of age instead of for all ages collectively. The lower fence was set at the first quartile minus 1.5 multiplied by the difference between the third and first quartiles, while the upper fence was set at the third quartile plus 1.5 multiplied by the difference between the third and first quartiles. After the fencing of outliers, linearity and heteroskedasticity were evaluated in pairwise plots which were followed by the Shapiro-Wilk test for normality [75]. Next, the homogeneity of regression slopes and homoscedasticity of residuals for all groups were evaluated via Levene’s test.

In order to test our hypotheses, an analysis of covariance (ANCOVA) was used to determine if there were any statistically significant group effects on EA-CSF volume. The following predictors and covariates were included in the model: diagnostic group, mean-centered age, mean-centered age^2^, scan site, sex, mean-centered eTIV, mean-centered eTIV^2^, a mean-centered age by group interaction, and a mean-centered age^2^ by group interaction. The estimated total intracranial volume covariates were not originally included in the preregistration and were included in the model after evaluating the need to control head size over age (given that we are interested in age effects on EA-CSF volume). Additionally, for the purposes of the cortical thickness analysis, total mean cortical thickness was operationalized as the sum of the mean cortical thicknesses from each hemisphere divided by two. All analyses were performed in R 4.2.0 [76], and the package car was used to perform the ANCOVA [77].

## Results

### Group, age, and other covariate effects on EA-CSF volume

In order to test the hypothesis that there are group differences in EA-CSF volume in children and adolescents, we used an ANCOVA. Among other assumptions, this statistical test assumes that the residuals are normally distributed, and variance is homogenous across all groups. We formally tested these two assumptions: the Shapiro-Wilk test for normality indicated that the residuals are not normally distributed (*W* = 0.99, *p* < .001), and Levene’s test indicated that there is homogeneity of variance for the three groups (*F*(2, 443) = 2.36, *p* = 0.09).

**Fig. 3:**
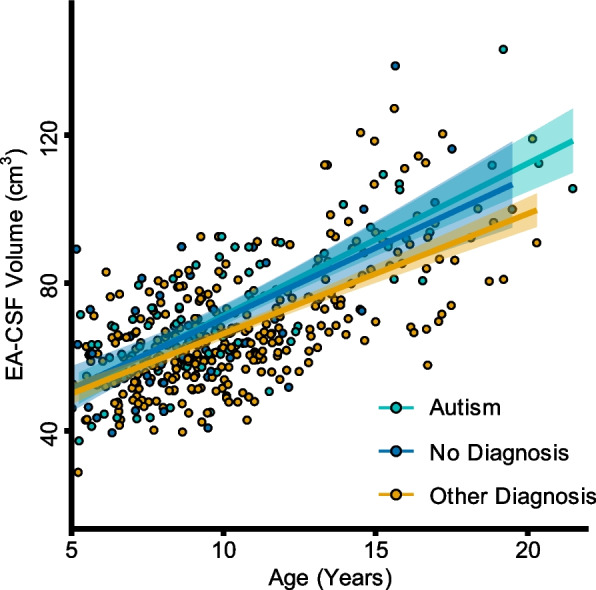
No significant difference in EA-CSF volumes between the ASD, No Diagnosis, and Other Diagnosis groups. Depicted is the linear trajectory of the EA-CSF volumes between 5 and 21 years. No significant difference between the three groups was found (group × age interaction*, F*(2, 433) = 0.34, *p* = 0.72, *η*^2^ = 0.001). A linear model was used to fit the developmental trajectories for each group. Each scan is represented by a circle

Following assumptions testing, the main ANCOVA analysis was performed. A significant effect of age on EA-CSF volume was found (*F*(1, 433) = 44.85, *p* < .001, *η*^2^ = 0.04) in addition to a significant eTIV effect (*F*(1, 433) = 132.56, *p* < .001, *η*^2^ = 0.12). This suggests that in this sample of children and adolescents, age and eTIV are contributors to EA-CSF volume. As demonstrated in Fig. 3, the relationship between age and EA-CSF volume is positive and linear. There was also a significant effect of the intercept (*F*(1, 433) = 1697.82, *p* < .001). Interestingly, there was neither a significant effect of diagnostic group on EA-CSF volume (*F*(2, 433) = 2.69, *p* = .07, *η*^2^ = 0.01), nor a significant group by age interaction (*F*(2, 433) = 0.34, *p* = 0.72, *η*^2^ = 0.001). Thus, contrary to our hypothesis, there is no difference in EA-CSF volume between the No Diagnosis, ASD, and Other Diagnosis groups. Additionally, no significant differences between groups were observed after re-centering age at 5 (*F*(2, 433) = 1.23, *p* = 0.29), 10 (*F*(2, 433) = 2.74, *p* = .07), 15 (*F*(2, 433) = 2.27, *p* = 0.11), and 20 (*F*(2, 433) = 1.95, *p* = 0.14) years.

Given the primary aim of this study in testing the specificity of potential differences in EA-CSF volume to ASD, a series of exploratory analyses comparing ASD and each other diagnosis included within the “Other Diagnosis” group were conducted. These consisted of multiple linear regressions with EA-CSF volume as the dependent variable, a binary group variable, and each of the covariates included in the main model as previously described. In order to control for comorbidity, autistic participants with a concurrent diagnosis of the specific diagnostic group being tested against were dropped from the analysis. No significant group differences in EA-CSF volume were found post-Bonferroni correction (α = .01) for the ADHD combined type (*t*(147) = 2.02, *p* = .05), ADHD inattentive type (*t*(163) = 2.08, *p* = .04), generalized anxiety disorder (*t*(122) = −1.84, *p* = .07), social anxiety disorder (*t*(114) = −1.85, *p* = .07), and specific learning disorder-reading (*t*(148) = −1.74, *p* = .08) analyses.

Differences in brain volume between ASD and neurotypical controls have been widely reported in the literature. However, to be sure that the choice to use eTIV covariates instead of total brain volume covariates did not change the overall outcome, a separate ANCOVA model was ran using total brain volume covariates substituted for the eTIV covariates. Similar to the reported results that included eTIV covariates, there was neither a significant effect of diagnostic group on EA-CSF volume (*F*(2, 433) = 2.76, *p* = .06, *η*^2^ = 0.01), nor a significant group by age interaction (*F*(2, 433) = 0.63, *p* = 0.53, *η*^2^ = 0.001).

### Sleep disturbances do not predict EA-CSF volume

**Fig. 4:**
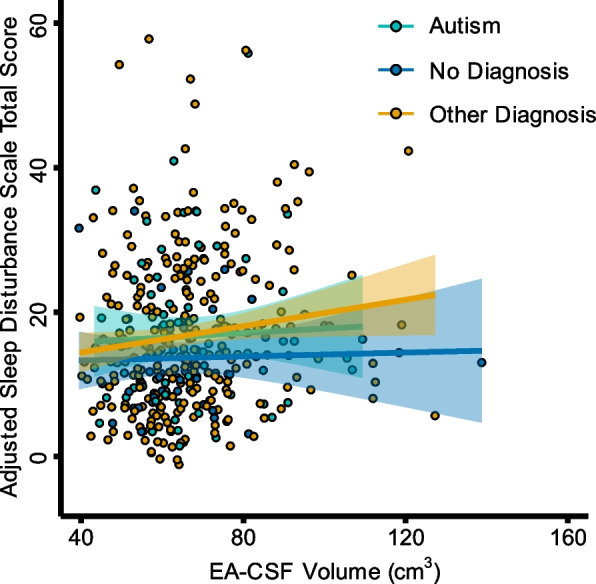
EA-CSF volumes were not a significant predictor of sleep disturbances in children and adolescents. Depicted is the lack of a relationship between EA-CSF volumes and the covariate-adjusted Sleep Disturbance Scale raw total score, (*t*(357) = 1.3, *p* = 0.19). Sleep Disturbance Scale total scores were adjusted by regressing out the effects of mean-centered age, mean-centered age^2^, diagnostic group (used within a dummy-coded framework where the ASD group was always the baseline), site, sex, mean-centered eTIV, and mean-centered eTIV^2^ using the following formula: SDS_adj_ = SDS_nat_ — [β_1_(mean-centered age_nat_ — mean of mean-centered age_nat_) + β_2_(mean-centered age^2^_nat_ — mean of mean-centered age^2^_nat_) + β_3_(group_nat_ — mean group_nat_) + β_4_(site_nat_ — mean site_nat_) + β_5_(sex_nat_ — mean sex_nat_) + β_6_(mean-centered eTIV_nat_ — mean of mean-centered eTIV_nat_) + β_7_(mean-centered eTIV_nat_ — mean of mean-centered eTIV_nat_)]. A linear model was used, and each scan is represented by a circle

In order to better understand the potential relationship between EA-CSF volume and sleep disturbances across later childhood and adolescence, an exploratory analysis not outlined in the preregistration was performed. Of the 446 T1w images included in the initial analysis, 367 participants ages 5.29–21.48 years (*M* = 9.94, *SD* = 2.7) had available Sleep Disturbances Scale scores and were included in this exploratory analysis. After assessing the normality of the residuals using the Shapiro-Wilk test, we employed a multiple linear regression, with Sleep Disturbances Scale raw total score as the dependent variable and the following predictors: EA-CSF volume, diagnostic group, mean-centered age, mean-centered age^2^, site, sex, mean-centered eTIV, and mean-centered eTIV^2^. EA-CSF volume was not a significant predictor of the Sleep Disturbances Scale raw total score (*t*(357) = 1.3, *p* = 0.19; see Fig. 4). However, there was a significant group difference in Sleep Disturbances Scale raw total scores between the No Diagnosis and ASD groups (*t*(357) = −2.33, *p* = .02). The intercept was also significant (*t*(357) = 9.57, *p* < .001). Thus, this evidence points to a lack of a relationship between EA-CSF volume and sleep disturbances in children and adolescents.

### Cortical thickness as a predictor of EA-CSF volume

**Fig. 5:**
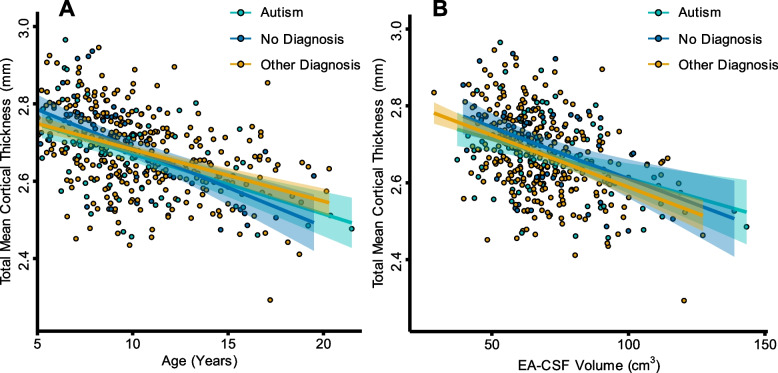
The relationship between cortical thickness, age, and EA-CSF volume. **A** depicts the linear trajectory of total mean cortical thickness across ages 5–21. A linear model was used to fit the trajectories, and each data point represents a single scan. **B** depicts the negative relationship between total mean cortical thickness and EA-CSF volume between 5 and 21 years. Cortical thickness was a significant predictor of EA-CSF volume (*t*(432) = -3.41, *p* < .001). A linear model was used to fit the trajectories, and each data point represents a single scan

Following the Peterson et al. [32] exploratory analysis examining the relationship between cortical thickness and EA-CSF volume, we aimed to replicate this analysis in a larger sample of children and adolescents. After normality assumptions testing (using the same steps outlined for the ANCOVA), a multiple linear regression was employed with EA-CSF volume as the dependent variable and the following predictors: total mean cortical thickness, diagnostic group, mean-centered age, mean-centered age^2^, site, sex, mean-centered eTIV, mean-centered eTIV^2^, a mean-centered age by group interaction, and a mean-centered age^2^ by group interaction. There appears to be a negative linear relationship between total mean cortical thickness and age such that total mean cortical thickness decreases as age increases (see Fig. 5A). A significant negative relationship between total mean cortical thickness and EA-CSF volume was found (*t*(432) = −3.41, *p* < .001), such that as the total mean cortical thickness estimate decreases, EA-CSF volume increases (see Fig. 5B).

## Discussion

In this study, we examined the specificity of an increased volume of EA-CSF to autism in a large sample of children and adolescents. Contrary to our hypothesis, we find no difference in EA-CSF volume between the ASD, Other Diagnosis, and No Diagnosis groups. Nevertheless, our results indicate that the ASD, Other Diagnosis, and No Diagnosis groups experience a linear increase in EA-CSF volume between the ages of 5 and 21. This trend is consistent with our previous findings [32] which indicated a doubling in EA-CSF volume between late childhood and adulthood. Additional analyses investigating the relationships between sleep disturbances and EA-CSF volume, and between cortical thickness and EA-CSF volume, were undertaken. No relationship was identified between EA-CSF volume and sleep disturbances. However, in a replication of Peterson et al. [32], a negative relationship between EA-CSF volume and cortical thickness was found.

### EA-CSF volume as a developmentally constrained potential biomarker

These results extend the work of Shen and colleagues using a saturated sample of children and adolescents with diverse neurodevelopmental and psychiatric conditions. Previously, EA-CSF volume in relation to ASD has been examined in infants 6–24 months old [29, 30] and children 2–4 years old [31]. Together, these studies indicate that an increased volume of EA-CSF may act as a biomarker or stratification marker for autism. As additional evidence for this idea, work undertaken by Murphy et al. [34] found no relationship between risk for schizophrenia and EA-CSF volume at ages 1 and 2. However, in a multi-cohort accelerated longitudinal design of participants 3–42 years old, no difference in EA-CSF volume between the ASD and typically developing control groups was found [32]. Similarly, the present study found no group difference in EA-CSF volume in participants 5–21 years old, suggesting once more that EA-CSF volume as a potential biomarker for ASD is likely constrained to a developmental period prior to age 5.

### Sleep disturbances and EA-CSF volume in ASD

In an exploratory analysis, we sought to better understand the relationship between sleep disturbances and EA-CSF volume. Previously, Shen et al. [31] identified a significant positive linear relationship between EA-CSF volume and sleep problems, such that greater sleep disturbances were associated with greater volumes of EA-CSF. Using the Sleep Disturbances Scale for Children, we were unable to reproduce the negative relationship described by Shen et al. [31] in this sample. This null result may be due to the combination of several factors, including differences in sample characteristics and sleep measures. Regardless, as this is an exploratory analysis, further investigation is needed to verify these results.

### Mechanisms for EA-CSF volume trajectories

As documented in the present study and Peterson et al. [32], a doubling in EA-CSF volume occurs across adolescence for participants of all diagnostic groups. One potential mechanism influencing this dramatic increase in EA-CSF volume is a decrease in cortical thickness. Such a decrease in mean cortical thickness is known to occur in adolescence via a synaptic pruning event [43–45]. As proposed in Peterson et al. [32], such a decrease in the volume of the cortical mantle would likely result in an increase in the space between the pial surface and dura mater, creating a deficit that could then be filled with an increased volume of EA-CSF. As preliminary evidence for this assertion, an exploratory analysis in Peterson et al. [32] found a negative relationship between mean cortical thickness and EA-CSF volume. Further evidence for this relationship was obtained in an analysis in the present study, which has the benefit of a larger sample of children and adolescents. Together, these two analyses point to a decrease in cortical thickness as a mechanism for an increased volume of EA-CSF. But beyond cortical thickness, other factors may be at play, particularly for ASD.

As a neurodevelopmental condition, ASD is associated with a variety of age-related organizational and structural aberrations in the brain. One well-documented finding in autism research is that of increased brain volume [2–5] and head size [6–9]. In a review on this subject, Ecker et al. [19] noted that the increased brain volume in autism typically resolves around ages 6 to 8. Similarly, in a longitudinal study, Aylward et al. [2] found that enlarged brain volume and head circumference in autism normalize by approximately age 12. However, results recently published by Lee et al. [78] indicate that, at least for their sample of participants 2–13 years of age, cerebral enlargement in ASD persists throughout childhood without normalization, with that enlargement largely occurring in boys with disproportionate megalencephaly. The timing of these findings concerning brain volume and head size appears to coincide with the normalization of EA-CSF volume in ASD, which may account for the lack of a group difference in EA-CSF volume reported here. However, the suggested incidence of total brain volume normalization occurring in conjunction with the normalization of EA-CSF volume has not been directly observed, and it remains to be seen if this relationship can be evidenced.

### Limitations and future directions

EA-CSF in this study was operationalized in accordance with previous work by Shen and colleagues in order to best extend their work. In congruence with that operationalization, we used the pipeline Auto EACSF, which has been found to be reliable with adult MRI scans [32]. And to ensure an accurate EA-CSF segmentation, the output for each image was visually quality controlled. However, this pipeline has only been used on MRI scans from adolescents and young adults in one prior study [32]. This could potentially be problematic since it is still unknown as to if the anterior commissure-posterior commissure plane retains the same distance relative to the top of the intracranial cavity across adolescence and into adulthood.

Also of note, ASD diagnoses in this study were determined using the computerized web-based version of the Schedule for Affective Disorders and Schizophrenia—Children’s version, which is not considered the gold standard for ASD diagnostics. While licensed clinicians oversaw the diagnostic process, the potential validity of the diagnoses given may have been affected. As a result, the Other Diagnosis group could include individuals with undiagnosed ASD, and the converse situation could be true as well. Ultimately, this limitation could affect the reported results and may be responsible for the null findings.

Additionally, it should be noted that the majority of this sample qualifies as having high verbal and cognitive performance, as defined using the criterion of a full-scale IQ score greater than or equal to 79 [51]. This may have accounted for the null result and limits the generalizability of these findings. In future investigations, researchers should consider examining the relationship between the level of verbal and cognitive performance and EA-CSF volume, particularly in autistic participants.

Finally, the greatest limitation of this study resides in the age range of the sample examined here. Previously, EA-CSF volume has been examined in infants and children 6 months to 4 years old. While our study expands this age range to encompass children and adolescents 5–21 years old, this does not allow for a direct comparison with previous research. Thus, future studies will need to elaborate upon this work by examining EA-CSF volume in younger participants of diverse neurodevelopmental and psychiatric conditions in order to determine if an increased volume of EA-CSF is specific to autism and for what timeframe.

## Conclusions

Inconsistent with our hypothesis, we found no group differences in EA-CSF volume in this cohort. These results indicate that an increased volume of EA-CSF is not specific to autistic individuals within the developmental period examined here.

## Data Availability

The data that support the findings of this study are not openly available due to reasons of sensitivity (e.g., human data) and are available from the Child Mind Institute upon completion of a data usage agreement. Phenotypic data may be accessed through the COllaborative Informatics and Neuroimaging Suite (COINS) Data Exchange upon completion of the data usage agreement (https://coins.trendscenter.org/). Neuroimaging data may be directly accessed through an Amazon Web Services S3 bucket (see http://fcon_1000.projects.nitrc.org/indi/cmi_healthy_brain_network/sharing_neuro.html#Direct%20Down).

## Abbreviations

ADHD: Attention-deficit hyperactivity disorder
ANCOVA: Analysis of covariance
ASD: Autism spectrum disorder
Auto EACSF: Automatic extra-axial cerebrospinal fluid
CSF: Cerebrospinal fluid
DSM-5: *Diagnostic and Statistical Manual of Mental Disorders, *Fifth Edition
EACSF: Extra-axial cerebrospinal fluid
eTIV: Estimated total intracranial volume
GAD: Generalized anxiety disorder
IQ: Intelligence quotient
MRI: Magnetic resonance imaging
WISC: Wechsler Intelligence Scale for Children
